# Clinically relevant chromosomal abnormalities and chromosomal polymorphisms in 7,530 patients undergoing reproductive genetic counseling: a retrospective cytogenetic study in a Chinese Han population

**DOI:** 10.3389/fgene.2026.1947419

**Published:** 2026-10-16

**Authors:** Songhang Wu, Hongyan Shang, Wenhua Wang, Dan Chen, Wennan Wu

**Affiliations:** 1 Fujian Key Laboratory of Laboratory Medicine, Department of Laboratory Medicine, Gene Diagnosis Research Center, Fujian Clinical Research Center for Clinical Immunology Laboratory Test, The First Affiliated Hospital of Fujian Medical University, Fuzhou, Fujian, China; 2 Department of Laboratory Medicine, National Regional Medical Center, Binhai Campus of the First Affiliated Hospital of Fujian Medical University, Fuzhou, Fujian, China

**Keywords:** chromosomal abnormalities, chromosomal polymorphism, correspondence analysis, genetic counseling, karyotype, reproductive disorder

## Abstract

**Background:**

Chromosomal aberrations are important causes of reproductive disorders, but their clinical interpretation is often complicated by the presence of benign chromosomal polymorphisms that may obscure the true diagnostic yield of karyotyping. The aim of this study was to evaluate peripheral blood karyotyping in patients undergoing reproductive genetic counseling and to distinguish clinically relevant chromosomal abnormalities from chromosomal polymorphisms.

**Methods:**

A total of 7,530 patients who visited the reproductive center and the obstetrics and gynecology genetic clinic of the First Affiliated Hospital of Fujian Medical University from January 2016 to December 2022 were included. Peripheral blood karyotyping was performed using lymphocyte culture, routine chromosome preparation, and G-banding. Cytogenetic findings were classified into clinically relevant chromosomal abnormalities and chromosomal polymorphisms. Chromosomal polymorphisms were analyzed separately and were not counted as the primary diagnostic outcome. Correspondence analysis was performed using an observed-count matrix linking major clinical manifestations with representative karyotypic findings.

**Results:**

A total of 552 abnormal cytogenetic findings were detected, corresponding to an overall cytogenetic finding rate of 7.33% (552/7,530). Among them, 246 cases were classified as clinically relevant chromosomal abnormalities, giving a primary diagnostic rate of 3.27% (246/7,530), whereas 306 cases were chromosomal polymorphisms, accounting for 4.06% of the total cohort and 55.43% of all abnormal cytogenetic findings. In correspondence analysis, the first two dimensions explained 77.9% and 15.2% of the total inertia, respectively. The global chi-square test indicated a non-random association between clinical manifestations and representative karyotypic findings (chi-square = 69.92, df = 16, P < 0.001).

**Conclusion:**

Peripheral blood karyotyping remains a useful first-line cytogenetic test in reproductive genetic counseling. Clinically relevant chromosomal abnormalities and chromosomal polymorphisms should be reported and interpreted separately. Phenotype-guided interpretation may improve the clinical value of karyotyping and help identify patients who require additional molecular confirmation or multidisciplinary counseling.

## 1 Introduction

Reproductive disorders are multifactorial conditions. Uterine structural abnormalities, endocrine imbalances, thrombophilia, environmental factors, and genetic factors may contribute to infertility, recurrent miscarriage, and repeated implantation failure (Crain et al., 2008; Ho et al., 2017). The global prevalence of infertility has been estimated at 8%–12% among couples of reproductive age, and population-based data from China have reported a substantial burden of infertility (Vander Borght and Wyns, 2018; Zhou et al., 2018).

Chromosomal aberrations are important causes of reproductive disorders. Patients with infertility, severe spermatogenic impairment, recurrent miscarriage, or developmental abnormalities of the reproductive system may have a higher likelihood of clinically relevant karyotypic findings than unselected populations (Caseiro et al., 2015; Guerri et al., 2019; Kuroda et al., 2020; Gunes and Esteves, 2021). Peripheral blood karyotyping remains a first-line cytogenetic method for detecting numerical abnormalities, balanced structural rearrangements, sex chromosome abnormalities, and other large-scale chromosomal changes (Gardner et al., 2012; Gonzales et al., 2016).

However, interpretation of karyotype results in reproductive genetic counseling is not always straightforward. Chromosomal polymorphisms, including inv(9), qh ± variants, and D/G group heteromorphisms, are commonly reported in cytogenetic laboratories, but their clinical significance remains controversial. Mixing these variants with clinically relevant abnormalities may overestimate the diagnostic yield of karyotyping. Therefore, this study separately analyzed clinically relevant chromosomal abnormalities and chromosomal polymorphisms in 7,530 patients undergoing reproductive genetic counseling, and explored phenotype-karyotype relationships using correspondence analysis.

## 2 Methods

### 2.1 Subjects

This was a retrospective observational study. A total of 7,530 reproductive genetic counseling patients who visited the reproductive center and the obstetrics and gynecology genetic clinic of the First Affiliated Hospital of Fujian Medical University from January 2016 to December 2022 were included in the final analysis. Patients were included if they were referred for infertility, severe spermatogenic impairment, menstrual or endocrine abnormalities, reproductive organ developmental abnormalities, adverse pregnancy outcomes, or reproductive genetic counseling. Patients with incomplete essential clinical or karyotype information, or a recorded history of radiation exposure, were excluded.

Clinical indications were assigned according to the referral diagnosis documented in the electronic medical record. Infertility was defined by the clinical referral diagnosis recorded by the reproductive or obstetrics and gynecology clinic. Oligoasthenospermia/azoospermia was based on the referring diagnosis supported by semen analysis. Hormonal abnormalities included recorded abnormalities in reproductive endocrine evaluation, including abnormalities involving follicle-stimulating hormone, luteinizing hormone, estradiol, progesterone, testosterone, prolactin, or other sex hormone results. Reproductive organ developmental abnormalities included congenital or clinically suspected developmental abnormalities of reproductive organs. Adverse pregnancy outcomes included recurrent miscarriage, embryo arrest, stillbirth, fetal abnormalities, or prior offspring with birth defects as recorded in the referral diagnosis. Genetic counseling indications referred to patients or couples seeking reproductive genetic risk assessment without a more specific categorized indication. The study was approved by the Ethics Committee of the First Affiliated Hospital of Fujian Medical University (Approval No. MTCA, ECFAH of FMU [2015]084-3). The study was conducted in accordance with the Declaration of Helsinki and applicable institutional and national ethical requirements. The participants provided written informed consent to participate in the study.

### 2.2 G-banding

Peripheral blood (3 mL) was collected into a sodium heparin anticoagulant tube under sterile conditions. Within 1 h, 0.5 mL of blood was inoculated into lymphocyte culture medium and incubated at 37 °C in a humidified atmosphere containing 5% CO2. After 72 h, colchicine was added at a final concentration of 20 μg/mL, and cells were harvested after an additional 2 h. Standard hypotonic treatment, fixation, slide preparation, and G-banding were performed. At least 20 metaphase cells were counted, and at least five metaphases were analyzed for each patient. For suspected mosaicism or confirmed abnormal karyotypes, the analysis was extended to a total of at least 30 metaphases when required, rather than the routine 20 metaphase count for normal samples. The analyzed chromosome resolution was greater than 320 bands. Karyotypes were described according to the International System for Human Cytogenomic Nomenclature (ISCN 2016) (McGowan-Jordan et al., 2016). Karyotype images were captured and analyzed using the Leica GSL120 automated chromosome scanning and image analysis system (Leica Microsystems, Germany).

### 2.3 Classification of cytogenetic findings

To avoid overestimation of diagnostic yield, cytogenetic findings were classified into two categories before analysis. Clinically relevant chromosomal abnormalities included autosomal numerical abnormalities, sex chromosome numerical abnormalities, autosomal structural abnormalities, sex chromosome structural abnormalities, sex reversal or disorders of sex development-related karyotypes, marker chromosomes, and other abnormalities with potential reproductive implications. Chromosomal polymorphisms included inv(9), 1/9/16qh ± variants, D/G group variants, inv(Y), Yqh±, and other heterochromatic variants. Chromosomal polymorphisms were analyzed separately because their clinical significance remains controversial and they were not counted as the primary clinically relevant diagnostic outcome.

### 2.4 Statistical analysis

Descriptive statistics were used to summarize the distribution of cytogenetic findings. The overall cytogenetic finding rate was calculated as total abnormal cytogenetic findings divided by all tested patients. The primary diagnostic rate was calculated as clinically relevant chromosomal abnormalities divided by all tested patients. Chromosomal polymorphisms were reported separately.

Correspondence analysis was performed in Python (version 3.11) using a singular value decomposition of the standardized residual matrix. The input matrix was constructed from observed counts linking major clinical manifestations to representative karyotypic categories. Each cell represented the number of patients with a specified clinical manifestation and karyotypic finding. Row and column principal coordinates were calculated and visualized in a two-dimensional biplot using matplotlib. Dimension-level inertia was calculated to quantify the proportion of the total association represented by each dimension. A chi-square test of independence was used to assess the global association between row and column categories. A P value less than 0.05 was considered statistically significant.

## 3 Results

### 3.1 Demographic characteristics and overall cytogenetic findings

The study included 7,530 patients aged 18–55 years, including 3,903 males and 3,627 females. Male patients were older than female patients [male vs. female: 31.32 ± 5.09 years vs. 29.65 ± 4.68 years, P < 0.001].

A total of 552 abnormal cytogenetic findings were detected, corresponding to an overall cytogenetic finding rate of 7.33% (552/7,530). After classification, 246 cases were clinically relevant chromosomal abnormalities, corresponding to a primary diagnostic rate of 3.27% (246/7,530). Chromosomal polymorphisms were identified in 306 cases, accounting for 4.06% of the total cohort and 55.43% of all abnormal cytogenetic findings. The overall classification is shown in Table 1.

**TABLE 1:** Overall classification of cytogenetic findings in the cohort.

Category	Included findings	Cases	Detection rate in total cohort (%)	Proportion among abnormal cytogenetic findings (%)	Interpretation
Clinically relevant chromosomal abnormalities	Numerical and structural abnormalities of autosomes and sex chromosomes, sex reversal/DSD-related karyotypes, marker chromosomes, and others^a^	246	3.27	44.57	Primary diagnostic outcome; interpreted according to phenotype and reproductive history
Chromosomal polymorphisms	inv(9), 1/9/16qh±, D/G group variants, inv(Y), Yqh±, and other heterochromatic variants	306	4.06	55.43	Analyzed separately because clinical significance remains controversial; not counted as the primary diagnostic outcome
Total abnormal cytogenetic findings	All findings above	552	7.33	100.00	Overall cytogenetic finding rate

Detection rate was calculated using the total cohort of 7,530 patients as the denominator. Chromosomal polymorphisms were analyzed separately and were not included in the primary clinically relevant diagnostic rate.

^a^ Includes autosomal numerical abnormalities, sex chromosome numerical abnormalities, autosomal structural abnormalities, sex chromosome structural abnormalities, sex reversal/disorders of sex development (DSD)-related karyotypes, marker chromosomes, and other findings with potential reproductive implications.

### 3.2 Descriptive distribution of clinically relevant chromosomal abnormalities

Among the 552 abnormal cytogenetic findings, sex chromosome numerical abnormalities accounted for 146 cases (1.94% of the total cohort; 26.45% of all abnormal cytogenetic findings), autosomal structural abnormalities for 83 cases (1.10%; 15.04%), autosomal numerical abnormalities for 7 cases (0.09%; 1.27%), sex chromosome structural abnormalities for 4 cases (0.05%; 0.72%), and sex reversal or disorders of sex development-related karyotypes for 6 cases (0.08%; 1.09%). Together, these categories constituted 246 clinically relevant chromosomal abnormalities (3.27% of the total cohort). Detailed distribution is shown in Table 2.

**TABLE 2:** Distribution of clinically relevant chromosomal abnormalities.

Category	Cases	Detection rate in total cohort (%)	Proportion among abnormal cytogenetic findings (%)	Representative clinical context
Autosomal numerical abnormalities	7	0.09	1.27	Miscarriage, birth defects, infertility-related indications
Sex chromosome numerical abnormalities	146	1.94	26.45	Azoospermia, oligozoospermia, amenorrhea, Turner syndrome-related features
Autosomal structural abnormalities	83	1.10	15.04	Balanced translocation, Robertsonian translocation, inversion
Sex chromosome structural abnormalities	4	0.05	0.72	Y chromosome structural abnormalities and X chromosome deletion
Sex reversal/DSD-related karyotypes	6	0.08	1.09	46,XX male; 46,XY female or related DSD findings
Total clinically relevant abnormalities	246	3.27	44.57	Primary diagnostic outcome

Proportion was calculated using all 552 abnormal cytogenetic findings as the denominator. Chromosomal polymorphisms are not included in this table.

### 3.3 Chromosomal polymorphisms

Chromosomal polymorphisms were identified in 306 cases. The most frequent variant was inv(9) (114 cases), accounting for 37.25% of chromosomal polymorphisms and 20.65% of all abnormal cytogenetic findings. This was followed by 1/9/16qh ± variants (87 cases; 28.43% of polymorphisms), D/G group variants (51 cases; 16.67%), Yqh± (37 cases; 12.09%), inv(Y) (14 cases; 4.58%), and rare variants including 19qh+, 10qh+, and inv(1). Detailed distribution of chromosomal polymorphisms is provided in Supplementary Table S1.

### 3.4 Age distribution

The highest overall cytogenetic finding rate was observed in the 26–30-year age group (8.09%), followed by the 18–25-year group (7.84%), 46–55-year group (7.58%), 41–45-year group (7.51%), 31–35-year group (6.53%), and 36–40-year group (5.91%). Because some age subgroups had small sample sizes, age-specific patterns should be interpreted descriptively.

### 3.5 Correspondence analysis

Correspondence analysis was performed using an observed-count matrix of major clinical manifestations and representative karyotypic findings. The matrix included 513 representative findings from major categories selected for phenotype-karyotype visualization (Table 3). The global chi-square test indicated a non-random association between clinical manifestations and representative karyotypic findings (chi-square = 69.92, df = 16, P < 0.001). The first two dimensions explained 77.9% and 15.2% of the total inertia, respectively, with a cumulative inertia of 93.1%.

**TABLE 3:** Observed-count matrix used for correspondence analysis.

Clinical manifestation	XXY-related karyotypes	45,X/mosaic Turner	Balanced translocation	Robertsonian translocation	Chromosomal polymorphisms
Oligoasthenospermia/azoospermia	49	4	10	3	40
Infertility	67	5	20	8	176
Adverse pregnancy outcomes	0	0	3	2	8
Irregular menstruation/amenorrhea	0	1	9	2	40
Genetic counseling	11	0	7	6	42

Cells are observed counts linking major clinical manifestations with representative karyotypic categories. The matrix included 513 representative findings and was used for correspondence analysis.

The two-dimensional biplot showed phenotype-karyotype correspondence patterns. Oligoasthenospermia/azoospermia was positioned close to XXY-related karyotypes, adverse pregnancy outcomes were positioned closer to balanced and Robertsonian translocations, and infertility was positioned near chromosomal polymorphisms. Categories located near the origin contributed less to discrimination along the first two dimensions than categories positioned farther from the origin. Because no bootstrap or permutation-based stability assessment was performed, the biplot should be interpreted as an exploratory visualization rather than a confirmatory clustering analysis (Figure 1).

**FIGURE 1:**
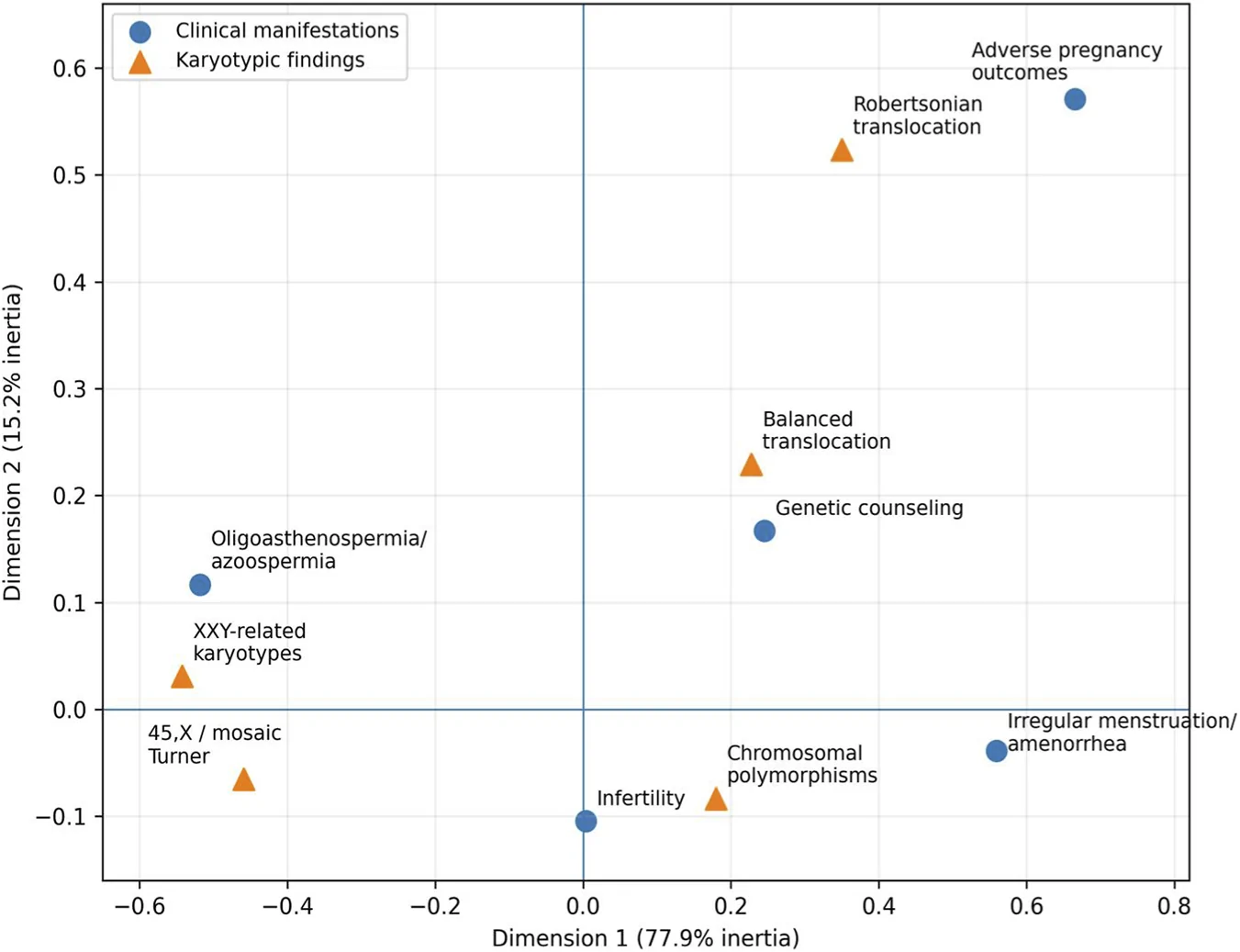
Correspondence analysis biplot of clinical manifestations and representative karyotypic findings. The first two dimensions explained 77.9% and 15.2% of the total inertia, respectively. Points located closer to each other indicate stronger correspondence in the analyzed matrix. The biplot was based on observed-count data and should be interpreted as an exploratory visualization of phenotype-karyotype patterns.

## 4 Discussion

In this retrospective observational study of 7,530 patients undergoing reproductive genetic counseling, we separated clinically relevant chromosomal abnormalities from chromosomal polymorphisms to avoid overestimating diagnostic yield. The overall cytogenetic finding rate was 7.33%, while the primary diagnostic rate for clinically relevant chromosomal abnormalities was 3.27%. This distinction is important because chromosomal polymorphisms accounted for more than half of all reported cytogenetic findings but should not be interpreted in the same manner as numerical abnormalities, structural rearrangements, sex reversal, or sex chromosome abnormalities.

The descriptive distribution of clinically relevant abnormalities suggests that sex chromosome numerical abnormalities and autosomal structural abnormalities are important cytogenetic categories in reproductive genetic counseling. XXY-related karyotypes, Turner syndrome-related karyotypes, X/Y structural abnormalities, marker chromosomes, and sex reversal or disorders of sex development-related karyotypes require phenotype-based interpretation and, when appropriate, additional molecular confirmation such as FISH, CMA, CNV-seq, NGS, SRY testing, or AZF microdeletion testing. These techniques serve complementary roles in the cytogenetic workup: FISH (fluorescence *in situ* hybridization) is used for rapid detection of specific chromosomal sequences and mosaicism; CMA (chromosomal microarray analysis) and CNV-seq (copy number variation sequencing) detect submicroscopic copy number variations; NGS (next-generation sequencing) enables comprehensive genomic analysis; SRY (sex-determining region Y) testing detects the Y-chromosomal gene critical for testicular development; and AZF (azoospermia factor) microdeletion testing identifies deletions in the AZFa, AZFb, and AZFc regions on the Y chromosome, which are important causes of male infertility (Wapner et al., 2012; Salehi et al., 2006; Sukumaran et al., 2013; Cioppi et al., 2021).

Correspondence analysis provided an exploratory visualization of the relationship between clinical manifestations and representative karyotypic findings. The close positioning of oligoasthenospermia/azoospermia and XXY-related karyotypes is biologically and clinically plausible, as Klinefelter syndrome is a well-known chromosomal cause of severe male infertility. Likewise, the correspondence of adverse pregnancy outcomes with balanced and Robertsonian translocations is consistent with the role of structural rearrangements in unbalanced gamete formation, miscarriage, embryo arrest, and offspring with chromosomal imbalance (Dutta et al., 2011; Zhang et al., 2018). However, because correspondence analysis was exploratory and no bootstrap stability analysis was performed, these patterns should be interpreted as phenotype-guided visualization rather than definitive causal evidence.

Chromosomal polymorphisms were common in this cohort. Similar observations have been reported in previous cytogenetic studies of infertile populations and reproductive medicine cohorts (Madon et al., 2005; Minocherhomji et al., 2009; Sahin et al., 2008; Li et al., 2020). Nevertheless, the clinical significance of inv(9), qh ± variants, D/G group variants, and other heterochromatic variants remains controversial. Many of these variants are also observed in phenotypically normal individuals. Therefore, they should be reported descriptively and interpreted together with clinical phenotype, reproductive history, partner karyotype, and embryo or products-of-conception genetic results when available. Assigning direct pathogenic causality to chromosomal polymorphisms without supporting clinical or molecular evidence should be avoided.

Based on our cohort findings, routine laboratory practice, and published reproductive genetics literature, we proposed a clinical interpretation and genetic counseling framework for representative karyotypic findings (Table 4). This framework is not intended to replace formal professional guidelines or multidisciplinary clinical decision-making. Rather, it provides a practical structure for laboratory geneticists, reproductive medicine specialists, obstetricians, and genetic counselors to communicate cytogenetic results, determine whether additional testing is needed, and identify findings that require individualized reproductive counseling. In multidisciplinary team practice, such a framework may help separate clinically actionable findings from variants of uncertain or controversial significance.

**TABLE 4:** Proposed clinical interpretation and genetic counseling framework for representative karyotypic findings.

Karyotypic finding	Main associated clinical features	Additional evaluation/testing to consider	Clinical/genetic counseling considerations
47,XXY/mosaic XXY	Azoospermia, severe oligoasthenospermia, male infertility	Andrological and endocrine evaluation; semen and fertility assessment; additional Y-chromosome testing when clinically indicated	Fertility counseling and individualized discussion of reproductive options, including assisted reproductive strategies when appropriate (Zitzmann et al., 2021)
Turner syndrome-related karyotypes	Amenorrhea, ovarian insufficiency, infertility, reproductive organ developmental abnormalities	Endocrine, cardiovascular, and reproductive assessment; additional genetic characterization when indicated by the karyotype and phenotype	Counseling regarding fertility potential, pregnancy-related risks, and individualized reproductive planning; multidisciplinary follow-up when appropriate (Gravholt et al., 2024)
Balanced or Robertsonian translocation	Recurrent miscarriage, adverse pregnancy outcomes, infertility	Partner karyotyping and individualized reproductive genetic assessment	Counseling regarding reproductive and recurrence risks; PGT-SR and prenatal diagnostic options may be discussed according to individual reproductive plans (ESHRE Guideline Group on RPL, 2023)
Marker chromosome	Azoospermia, infertility, developmental abnormalities, or uncertain phenotype	Further characterization by FISH and/or genomic copy-number analysis when the origin or genomic content cannot be adequately defined by conventional karyotyping	Interpretation should consider marker origin, genomic content, mosaicism, phenotype, and inheritance; multidisciplinary evaluation may be appropriate in complex cases (Yu et al., 2012; Joksic et al., 2024)
Sex reversal/DSD-related karyotypes	Infertility, azoospermia, amenorrhea, gonadal dysgenesis	Endocrine evaluation, imaging, biochemical assessment, and phenotype-directed genetic testing, including SRY or other targeted molecular analyses when appropriate	Multidisciplinary evaluation involving clinical genetics, endocrinology, urology/andrology, and/or gynecology as appropriate; individualized genetic and psychosocial counseling (Lee et al., 2016; Cools et al., 2018)
X/Y chromosome structural abnormalities	Amenorrhea, azoospermia, oligozoospermia, infertility	Phenotype-directed endocrine and molecular evaluation; Y-chromosome/AZF testing in males with azoospermia or severe oligozoospermia when appropriate	Reproductive counseling should be individualized according to the specific structural abnormality, phenotype, reproductive implications, and potential transmission risk (Krausz et al., 2024; Gravholt et al., 2024)
Chromosomal polymorphisms	Infertility or adverse reproductive history, but also observed in phenotypically normal individuals	No reflex molecular testing solely on the basis of a chromosomal polymorphism; further evaluation should be guided by phenotype, reproductive history, and other clinical findings	Interpret cautiously and avoid assigning direct pathogenic causality without supportive phenotypic, familial, reproductive, or molecular evidence (Ralapanawe et al., 2023; Li et al., 2020)

This proposed framework was developed based on the findings of the present cohort and published clinical guidelines, consensus statements, and reproductive genetics literature. Additional evaluation or testing should be considered according to the specific karyotypic finding, clinical phenotype, and reproductive history. The framework is intended to support laboratory interpretation, multidisciplinary referral, and genetic counseling and should not replace formal professional guidelines or individualized clinical decision-making. AZF, azoospermia factor; DSD, differences/disorders of sex development; FISH, fluorescence *in situ* hybridization; PGT-SR, preimplantation genetic testing for structural rearrangements; SRY, sex-determining region Y.

This study has several limitations. First, it was a single-center retrospective study, and selection bias could not be avoided. Second, clinical indication definitions were based on referral diagnoses recorded in the medical record; detailed standardized definitions, disease duration, and complete hormone profiles were not available for every patient. Third, complete partner karyotypes, longitudinal reproductive outcomes, and embryo or products-of-conception genetic results were not available for all patients, limiting our ability to directly evaluate the reproductive impact of specific findings, especially chromosomal polymorphisms. Fourth, correspondence analysis was based on representative karyotype categories and should be regarded as exploratory. Finally, G-banding has inherent resolution limitations and cannot reliably detect submicroscopic copy-number variants or single-gene defects. Therefore, conventional karyotyping should be regarded as complementary to, rather than competitive with, molecular genomic testing. When clinically indicated, additional testing such as CMA/CNV-seq or NGS should be considered to identify abnormalities beyond the resolution of conventional cytogenetics.

Conventional karyotyping and contemporary genomic technologies should be regarded as complementary rather than competing diagnostic approaches in reproductive genetics. G-banded karyotyping provides a genome-wide overview of chromosome number and large-scale structure and remains particularly useful for detecting aneuploidies, balanced translocations, Robertsonian translocations, large inversions, sex chromosome abnormalities, and chromosomal mosaicism. Its major limitation is relatively low resolution, which prevents reliable detection of submicroscopic copy-number variants and single-gene defects. In contrast, CMA and CNV-seq provide higher resolution for detecting submicroscopic copy-number alterations but generally cannot reliably identify balanced chromosomal rearrangements. NGS is particularly valuable for identifying pathogenic variants underlying single-gene disorders but does not replace chromosome-level evaluation when numerical or structural abnormalities are suspected. OGM is an emerging high-resolution technology capable of detecting a broad spectrum of balanced and unbalanced structural variants, although its availability, standardization, and clinical validation in reproductive medicine remain evolving (Chebly, 2026). Therefore, conventional karyotyping remains particularly important when aneuploidy, balanced rearrangements, chromosomal mosaicism, or large structural abnormalities are suspected.

Looking ahead, the integration of conventional karyotyping with emerging genomic technologies will likely shape the future of reproductive genetic diagnosis. A phenotype-guided, tiered approach—using karyotyping when chromosomal numerical or structural abnormalities are suspected, followed by CMA/CNV-seq for submicroscopic CNVs and NGS for suspected single-gene disorders—may improve diagnostic comprehensiveness while maintaining clinical practicality. OGM may further expand the detection of structural variants as its clinical standardization and validation progress. Artificial intelligence-assisted karyotype analysis may also improve analytical efficiency in high-volume laboratories. Ultimately, future reproductive genetic diagnosis is likely to integrate conventional cytogenetics and molecular genomic technologies according to the clinical phenotype and suspected genetic mechanism.

In conclusion, peripheral blood karyotyping remains a useful first-line cytogenetic test in reproductive genetic counseling. Clinically relevant chromosomal abnormalities and chromosomal polymorphisms should be reported and interpreted separately. The primary diagnostic rate for clinically relevant abnormalities was 3.27%, whereas chromosomal polymorphisms accounted for 4.06% of the cohort. Phenotype-guided interpretation, supported by correspondence analysis and an explicitly defined counseling framework, may improve communication between cytogenetic laboratories and reproductive medicine teams. Additional molecular testing should be considered when conventional karyotyping is insufficient for clinical interpretation.

## Data Availability

The datasets analyzed in this study are not publicly available due to patient privacy and ethical restrictions. De-identified data may be made available by the corresponding author upon reasonable request, subject to institutional approval and applicable ethical requirements.

## References

[B1] Caseiro A. L. Regalo A. Pereira E. Esteves T. Fernandes F. Carvalho J. (2015). Implication of sperm chromosomal abnormalities in recurrent abortion and multiple implantation failure. Reprod. Biomed. Online 31, 481–485. 10.1016/j.rbmo.2015.07.001 26299791

[B2] Chebly A. (2026). Cytogenetics in the genomics era: why karyotyping still matters. BMC Med. Genomics 19, 10. 10.1186/s12920-025-02296-0 41535841 PMC12801557

[B3] Cioppi F. Rosta V. Krausz C. (2021). Genetics of azoospermia. Int. J. Mol. Sci. 22, 3264. 10.3390/ijms22063264 33806855 PMC8004677

[B4] Cools M. Nordenström A. Robeva R. Hall J. Westerveld P. Flück C. (2018). Caring for individuals with a difference of sex development (DSD): a consensus statement. Nat. Rev. Endocrinol. 14, 415–429. 10.1038/s41574-018-0010-8 29769693 PMC7136158

[B5] Crain D. A. Janssen S. J. Edwards T. M. Heindel J. Ho S. M. Hunt P. (2008). Female reproductive disorders: the roles of endocrine-disrupting compounds and developmental timing. Fertil. Steril. 90, 911–940. 10.1016/j.fertnstert.2008.08.067 18929049 PMC4086418

[B6] Dutta U. R. Rajitha P. Pidugu V. K. Dalal A. B. (2011). Cytogenetic abnormalities in 1162 couples with recurrent miscarriages in southern region of India: report and review. J. Assist. Reprod. Genet. 28, 145–149. 10.1007/s10815-010-9492-6 20931274 PMC3059523

[B7] ESHRE Guideline Group on RPL, Bender Atik R. Christiansen O. B. Elson J. Kolte A. M. Lewis S. (2023). ESHRE guideline: recurrent pregnancy loss: an update in 2022. Hum. Reprod. Open 2023 (1), hoad002. 10.1093/hropen/hoad002 36873081 PMC9982362

[B8] Gardner R. J. M. Sutherland G. R. Shaffer L. G. (2012). Chromosome Abnormalities and Genetic Counseling. 4th Edn. New York, NY: Oxford University Press.

[B9] Gonzales P. R. Carroll A. J. Korf B. R. (2016). Overview of clinical cytogenetics. Curr. Protoc. Hum. Genet. 89 (8.1.1–8.1.13), 1–8. 10.1002/0471142905.hg0801s89 27037488

[B10] Gravholt C. H. Andersen N. H. Christin-Maitre S. Davis S. M. Duijnhouwer A. Gawlik A. (2024). Clinical practice guidelines for the care of girls and women with Turner syndrome. Eur. J. Endocrinol. 190, G53–G151. 10.1093/ejendo/lvae050 38748847 PMC11759048

[B11] Guerri G. Maniscalchi T. Barati S. Gerli S. Di Renzo G. C. Della Morte C. (2019). Non-syndromic monogenic female infertility. Acta Biomed. 90, 68–74. 10.23750/abm.v90i10-S.8763 31577258 PMC7233646

[B12] Gunes S. Esteves S. C. (2021). Role of genetics and epigenetics in male infertility. Andrologia 53, e13586. 10.1111/and.13586 32314821

[B13] Ho S. M. Cheong A. Adgent M. A. Veevers J. Suen A. A. Tam N. N. C. (2017). Environmental factors, epigenetics, and developmental origin of reproductive disorders. Reprod. Toxicol. 68, 85–104. 10.1016/j.reprotox.2016.07.011 27421580 PMC5233640

[B14] Joksic I. Toljic M. Milacic I. Stankovic A. Karadzov Orlic N. Mikovic Z. (2024). Small supernumerary marker chromosomes in prenatal diagnosis—molecular characterization and clinical outcomes. Front. Genet. 14, 1326985. 10.3389/fgene.2023.1326985 38259626 PMC10800731

[B15] Krausz C. Navarro-Costa P. Wilke M. Tüttelmann F. (2024). EAA/EMQN best practice guidelines for molecular diagnosis of Y-chromosomal microdeletions: state of the art 2023. Andrology 12, 487–504. 10.1111/andr.13514 37674303

[B16] Kuroda S. Usui K. Sanjo H. Takeshima T. Kawahara T. Uemura H. (2020). Genetic disorders and male infertility. Reprod. Med. Biol. 19, 314–322. 10.1002/rmb2.12336 33071633 PMC7542010

[B17] Lee P. A. Nordenström A. Houk C. P. Ahmed S. F. Auchus R. Baratz A. (2016). Global disorders of sex development update since 2006: perceptions, approach and care. Horm. Res. Paediatr. 85, 158–180. 10.1159/000442975 26820577

[B18] Li S. J. Cheng Y. X. Ye S. Zhou D. N. Zhang Y. Yin T. L. (2020). Chromosomal polymorphisms associated with reproductive outcomes after IVF-ET. J. Assist. Reprod. Genet. 37, 1703–1710. 10.1007/s10815-020-01793-8 32451813 PMC7376992

[B19] Madon P. F. Athalye A. S. Parikh F. R. (2005). Polymorphic variants on chromosomes probably play a significant role in infertility. Reprod. Biomed. Online 11, 726–732. 10.1016/s1472-6483(10)61691-4 16417737

[B20] McGowan-Jordan J. Simons A. Schmid M. (2016). ISCN 2016: An International System for Human Cytogenomic Nomenclature (Basel: Karger).

[B21] Minocherhomji S. Athalye A. S. Madon P. F. Kulkarni D. Uttamchandani S. A. Parikh F. R. (2009). A case-control study identifying chromosomal polymorphic variations as forms of epigenetic alterations associated with the infertility phenotype. Fertil. Steril. 92, 88–95. 10.1016/j.fertnstert.2008.05.071 18692838

[B22] Ralapanawe M. S. B. Khattak H. Hapangama H. R. Weerakkody G. R. Papadopoulou A. Gallos I. (2023). Chromosomal polymorphisms in assisted reproduction: a systematic review and meta-analysis. Hum. Fertil. (Camb.) 26, 687–698. 10.1080/14647273.2022.2051614 35322731

[B23] Sahin F. I. Yilmaz Z. Yuregir O. O. Bulakbasi T. Ozer O. Zeyneloglu H. B. (2008). Chromosome heteromorphisms: an impact on infertility. J. Assist. Reprod. Genet. 25, 191–195. 10.1007/s10815-008-9216-3 18461436 PMC2582066

[B24] Salehi L. B. Scarciolla O. Vanni G. F. Nardone A. M. Frajese G. Novelli G. (2006). Identification of a novel mutation in the SRY gene in a 46,XY female patient. Eur. J. Med. Genet. 49, 494–498. 10.1016/j.ejmg.2006.03.003 16675314

[B25] Sukumaran A. Desmangles J. C. Gartner L. A. Buchlis J. (2013). Duplication of dosage sensitive sex reversal area in a 46,XY patient with normal sex determining region of Y causing complete sex reversal. J. Pediatr. Endocrinol. Metab. 26, 775–779. 10.1515/jpem-2012-0354 23612644

[B26] Vander Borght M. Wyns C. (2018). Fertility and infertility: definition and epidemiology. Clin. Biochem. 62, 2–10. 10.1016/j.clinbiochem.2018.03.012 29555319

[B27] Wapner R. J. Martin C. L. Levy B. Ballif B. C. Eng C. M. Zachary J. M. (2012). Chromosomal microarray versus karyotyping for prenatal diagnosis. N. Engl. J. Med. 367, 2175–2184. 10.1056/NEJMoa1203382 23215555 PMC3549418

[B28] Yu S. Fiedler S. D. Brawner S. J. Joyce J. M. Zhou X. G. Liu H. Y. (2012). Characterizing small supernumerary marker chromosomes with combination of multiple techniques. Cytogenet. Genome Res. 136, 6–14. 10.1159/000334271 22123409

[B29] Zhang T. Sun Y. Chen Z. Li T. (2018). Traditional and molecular chromosomal abnormality analysis of products of conception in spontaneous and recurrent miscarriage. BJOG 125, 414–420. 10.1111/1471-0528.15052 29220118

[B30] Zhou Z. Zheng D. Wu H. Li R. Xu S. Kang Y. (2018). Epidemiology of infertility in China: a population-based study. BJOG 125, 432–441. 10.1111/1471-0528.14966 29030908

[B31] Zitzmann M. Aksglaede L. Corona G. Isidori A. M. Juul A. T'Sjoen G. (2021). European Academy of Andrology guidelines on Klinefelter syndrome endorsing organization: European society of endocrinology. Andrology 9, 145–167. 10.1111/andr.12909 32959490

