# Supplementary material for: Clinically relevant chromosomal abnormalities and chromosomal polymorphisms in 7,530 patients undergoing reproductive genetic counseling: a retrospective cytogenetic study in a Chinese Han population
Source: Front Genet. 2026 Oct 16;17:1947419. doi: 10.3389/fgene.2026.1947419 (PMC13720713; doi:10.3389/fgene.2026.1947419)
Supplement: Supplementary file 1 [file Table1.docx]

**Supplementary Table S1. Distribution and clinical manifestations of chromosomal polymorphic variants**

| Chromosomal polymorphic variant | Cases | Detection rate in total cohort (%) | Proportion among abnormal cytogenetic findings (%) | Proportion among polymorphisms (%) | Main clinical manifestations |
| --- | --- | --- | --- | --- | --- |
| 1/9/16qh± | 87 | 1.16 | 15.76 | 28.43 | Infertility (51), irregular menstruation/amenorrhea (17), genetic counseling (13), oligoasthenospermia/azoospermia (5), adverse pregnancy outcomes (1) |
| D/G group variants | 51 | 0.68 | 9.24 | 16.67 | Infertility (28), irregular menstruation/amenorrhea (8), genetic counseling (7), oligoasthenospermia/azoospermia (5), adverse pregnancy outcomes (3) |
| inv(9) | 114 | 1.51 | 20.65 | 37.25 | Infertility (67), genetic counseling (18), irregular menstruation/amenorrhea (14), oligoasthenospermia/azoospermia (11), adverse pregnancy outcomes (4) |
| inv(Y) | 14 | 0.19 | 2.54 | 4.58 | Infertility (10), oligoasthenospermia/azoospermia (4) |
| Yqh± | 37 | 0.49 | 6.70 | 12.09 | Infertility (18), oligoasthenospermia/azoospermia (15), genetic counseling (4) |
| 19qh+ | 1 | 0.01 | 0.18 | 0.33 | Infertility (1) |
| 10qh+ | 1 | 0.01 | 0.18 | 0.33 | Irregular menstruation/amenorrhea (1) |
| inv(1) | 1 | 0.01 | 0.18 | 0.33 | Infertility (1) |
| Total | 306 | 4.06 | 55.43 | 100.00 | - |

Note: Chromosomal polymorphisms were analyzed separately because their clinical significance remains controversial. They were not counted as the primary clinically relevant diagnostic outcome.
